# Oocyte Vitrification for Fertility Preservation in Women with Benign Gynecologic Disease: French Clinical Practice Guidelines Developed by a Modified Delphi Consensus Process

**DOI:** 10.3390/jcm10173810

**Published:** 2021-08-25

**Authors:** Blandine Courbiere, Enora Le Roux, Emmanuelle Mathieu d’Argent, Antoine Torre, Catherine Patrat, Christophe Poncelet, Jacques Montagut, Anne-Sophie Gremeau, Hélène Creux, Maëliss Peigné, Isabella Chanavaz-Lacheray, Lara Dirian, Xavier Fritel, Jean-Luc Pouly, Arnaud Fauconnier, on behalf of the PreFerBe Expert Panel

**Affiliations:** 1 Department of Gynecology-Obstetric and Reproductive Medicine, AP-HM, Hôpital La Conception, 13005 Marseille, France; 2 Aix-Marseille Université, IMBE, CNRS, IRD, Avignon Université, 13005 Marseille, France; 3 Unité d’Epidémiologie Clinique, Hôpital Universitaire Robert Debré, AP-HP Nord-Université de Paris, Inserm, CIC 1426, 75019 Paris, France; enora.leroux2@aphp.fr; 4 ECEVE UMR 1123, Université de Paris, Inserm, 75019 Paris, France; 5 Department of Gynecology-Obstetric and Reproductive Medicine, GRC6-UPMC, Centre Expert en Endométriose (C3E), Université Pierre-et-Marie-Curie Paris 6, Hôpital Tenon, CHU de Tenon, AP-HP, 4, rue de la Chine, 75020 Paris, France; emmanuelle.mathieu@aphp.fr; 6 Department of Gynecology-Obstetric and Reproductive Medicine, CHU Rouen, 37 bd Gambetta, 76000 Rouen, France; antoine.torre@chu-rouen.fr; 7 Service de Biologie de la Reproduction—CECOS, APHP Centre—Université de Paris, Site Cochin, Inserm U1016, 75014 Paris, France; catherine.patrat@aphp.fr; 8 Department of Gynecology-Obstetric, UFR SMBH Leonard de Vinci, CH René Dubos, 95000 Cergy-Pontoise, France; christophe.poncelet@ght-novo.fr; 9 Université Sorbonne Paris Nord—Paris 13, 93200 Saint-Denis, France; maeliss.peigne@aphp.fr; 10 Institut Francophone de Recherche et d’Etudes Appliquées à la Reproduction, Ifreares Toulouse, 31000 Toulouse, France; j.montagut@wanadoo.fr; 11 Department of Gynecologic Surgery and IVF, Clermont-Ferrand, University Hospital Clermont-Ferrand, 63000 Clermont-Ferrand, France; asgremeau@chu-clermontferrand.fr; 12 Clinique Saint Roch, Department of Gynecology-Obstetric and Reproductive Medicine, 34000 Montpellier, France; helenecreux@gmail.com; 13 Department of Reproductive Medicine and Fertility Preservation, AP-HP Hôpital Jean Verdier, 93143 Bondy, France; 14 Centre d’Endométriose, Clinique Tivoli Ducos, 33000 Bordeaux, France; isabella.chanavaz.lacheray@gmail.com; 15 EndoFrance, Association Française de lutte contre l’Endométriose, 70190 Tresilley, France; dirian.lara@gmail.com; 16 Department of Gynecology-Obstetric and Reproductive Medicine, CHU Poitiers, 86000 Poitiers, France; xavier.fritel@chu-poitiers.fr; 17 Inserm CIC-P 1402, 86021 Poitiers, France; 18 Department of Gynecology-Obstetric, CH Moulins Yzeure, 03000 Moulins, France; pouly.jean@orange.fr; 19 Department of Gynecology and Obstetrics, CHI Poissy-Saint-Germain-en Laye, 78300 Poissy, France; arnaud.fauconnier@ght-yvelinesnord.fr; 20 Research Unit 7285 Risk and Safety in Clinical Medicine for Women and Perinatal Health, Paris-Saclay University, 78300 Poissy, France

**Keywords:** fertility preservation, oocyte vitrification, benign gynecologic disease, modified Delphi method, consensus study

## Abstract

International guidelines are published to provide standardized information and fertility preservation (FP) care for adults and children. The purpose of the study was to conduct a modified Delphi process for generating FP guidelines for BGD. A steering committee identified 42 potential FP practices for BGD. Then 114 key stakeholders were asked to participate in a modified Delphi process via two online survey rounds and a final meeting. Consensus was reached for 28 items. Among them, stakeholders rated age-specific information concerning the risk of diminished ovarian reserve after surgery as important but rejected proposals setting various upper and lower age limits for FP. All women should be informed about the benefit/risk balance of oocyte vitrification—in particular about the likelihood of live birth according to age. FP should not be offered in rASRM stages I and II endometriosis without endometriomas. These guidelines could be useful for gynecologists to identify situations at risk of infertility and to better inform women with BGDs who might need personalized counseling for FP.

## 1. Introduction

International guidelines for clinical practice are published in oncology to offer standardized information and fertility preservation (FP) care for adults and children with cancer [1]. The ESHRE Guideline Group on Female Fertility Preservation has recently published recommendations on information provision and support, pre-FP assessment, FP intervention and after treatment care for patients diagnosed with: cancer and benign diseases undergoing gonadotoxic treatments, transgender men, and women requesting oocyte cryopreservation for age-related fertility loss [2]. A few additional clinical practice guidelines have been regarding non-oncological indications, but most recommendations are based primarily on data coming from oncofertility studies or expert opinion rather than studies with a stronger and broader evidence base [3,4,5]. The aim of a Delphi consensus is to provide expert answers in addition to the already existing recommendations when the data in the literature are still insufficient to issue recommendations with a high level of evidence. However, the growing literature in this field should provide stronger data in the future [6].

FP is inscribed in the law of several European countries: any man, woman, or child may have their gametes or germinal tissue collected and cryopreserved when a necessary medical treatment is likely impairing their fertility, or when this fertility is at risk of premature impairment. Some government health insurance programs cover most or all of the costs associated with FP for medical reasons. Because FP is free of charge for all patients in France, its indications may be enlarged. As both physicians and citizens, we have a responsibility to think about the cost-effectiveness and the cost–benefit balance of a FP strategy for benign gynecologic disease (BGD). Moreover, physicians need help in their everyday clinical practice to selecting appropriate indications for FP.

Given the lack of published evidence about indications for BGD, the steering committee of this study chose to address a wide set of questions to an expert panel for their opinion. We conducted a modified Delphi process with native European French-speaking experts, aimed at generating clinical guidelines about (i) the information to be provided to women of reproductive age with a BGD, (ii) technical aspects of FP for BGD, (iii) the indications for FP in endometriosis, (iv) the indications for FP in non-endometriosis BGD, and (v) the indications for FP after a fortuitous diagnosis of an idiopathic diminished ovarian reserve.

## 2. Materials and Methods

We conducted a modified Delphi consensus process with two online survey rounds and a final meeting among a multidisciplinary expert panel comprising gynecologists specialized in reproductive medicine, gynecologic surgeons, embryologists, and women with personal experience in the fields of infertility, endometriosis, or female fertility preservation. Briefly, the modified Delphi process is a recognized technique used to develop quality indicators in healthcare. It involves reaching a consensus after performing several rounds of questionnaires that collect expert opinions of clinical or scientific evidence. To avoid performing too many online rounds, we followed the methodology and guidance for the modified Delphi method as described by Boulkedid et al. (2011) [7].

### 2.1. Preselection of Statements and Delphi Questionnaire Preparation

The French national college of gynecologists and obstetricians (College National des Gynécologues et Obstétriciens Français (CNGOF)) designated a steering committee of 14 professionals based on their recognized expertise in reproductive medicine, endometriosis, gynecology, embryology, and fertility preservation. The committee also included a woman, with lived experience of endometriosis and infertility as a representative of a patient group (EndoFrance). This committee identified potentially relevant topics about FP for BGD, based on the international literature and their own experience, and chose to exclude from this survey oncological FP indications as well as autoimmune and endocrinologic diseases for which either the disease itself or its treatment might impair fertility.

### 2.2. Expert Panel Composition

To form a relevant expert panel, the steering committee aimed to gather a diverse group to ensure the broadest spectrum of opinion. The healthcare professionals were well-known French-speaking experts in infertility, including physicians specialized in reproductive medicine, gynecologic surgeons, obstetricians, embryologists, and specialists in pelvic imaging. They were selected from different geographic regions throughout France, Belgium, Switzerland, and the United Kingdom (UK) and, to ensure that they represent a wide array of clinical approaches, practices, and backgrounds, they practice medicine in teaching hospitals, general hospitals, or private hospitals and clinics. They were also selected to represent a broad range of age and experience levels. Expert patients were volunteers and came from two main French patient networks: one representing women with endometriosis (ENDOFRANCE https://www.endofrance.org (accessed on 18 August 2021)) and one representing infertile men and women (Association Collectif BAMP, https://bamp.fr (accessed on 18 August 2021)). We planned to include at least 10 panelists by stakeholder category. The expert panel was not remunerated for their participation.

### 2.3. Delphi Round 1

Panelists who had agreed to participate received an email link to access the self-administered questionnaire on a dedicated website. Non-responders were recontacted by email and telephone to encourage them to respond. Each panelist was asked to rate the 42 statements for agreement. Each item was rated on a 9-point scale, where 1 meant definitely disagree (not a relevant or appropriate practice) and 9 definitely agree (relevant and appropriate practice) with the statements. At the end of each of the five topics, the expert was invited to comment on the statements and suggest possible additional statements not included in the list.

Each statement was scored by its median. Statements were retained for the second round if the median score was 7, 8, or 9 and if at least 65% of the panel ratings were at least 7. At the end of the first round, the steering committee modified the questionnaire, adding, changing, or deleting some statements in accordance with the panelists’ votes, comments, and suggestions.

### 2.4. Delphi Round 2

The second round of self-administered questionnaire was sent by email to each expert who had participated in the first round. These panelists also received feedback on the first-round results (median panel rating, frequency distribution, and their own individual ratings). They were asked to re-rate each statement based on both their own opinion and the panel responses during the first round. To be included in the final set, statements had to have median ratings of 7–9 and 75% agreement among panelists [7].

### 2.5. Final Meeting for Approval of Selected Clinical Guidelines

The project concluded with a final meeting on 17 November 2020. Due to the COVID-19 pandemic, the meeting took place by videoconference. All panel members were invited for this consensus meeting, during which an overview of the results of the second-round ratings was reported, including the overall medians and the percentages of agreement. The meeting was chaired by three of the authors (B.C., E.L.R., and A.F.). This meeting enabled the clarification or rephrasing of some of the already accepted statements.

### 2.6. Statistical Analysis

We conducted a descriptive analysis of the participants’ characteristics and the data of each Delphi round. Results were reported as medians (Q1, Q3) for continuous variables and as frequency counts and percentages (%) for categorical variables. Medians and interquartile ranges during the Delphi rounds describe the relevance of each item, and percentages the agreement among panelists. Statistical analysis was conducted with SAS^®^ software v9.4 (SAS Institute Inc.; Cary, NC, USA).

This study did not require ethics review or approval by a research ethics committee as, consistent with European regulations, France does not require this approval for research based on questionnaires and interviews with health professionals (https://www.legifrance.gouv.fr/eli/decret/2017/5/9/AFSP1706303D/jo/texte (accessed on 18 August 2021)).

## 3. Results

### 3.1. Selection of Statements

The steering committee chose 42 statements to present for the first Delphi round. These statements were distributed into five general categories: (i) information to provide to women of reproductive age with a BGD (*n* = 9), (ii) technical aspects of FP for BGD (*n* = 6), (iii) indications for FP in endometriosis (*n* = 13), (iv) indications for FP for non-endometriosis BGD (*n* =10), and (v) indications for FP after the fortuitous diagnosis of an idiopathic diminished ovarian reserve (*n* = 4).

### 3.2. Composition of the Expert Panels

Overall, 114 experts were approached to participate in this modified Delphi procedure. Table 1 summarizes the characteristics of the panelists who responded, completing at least one questionnaire: 80 professionals and 6 patients.

#### 3.2.1. Delphi Round 1

**Figure 1:**
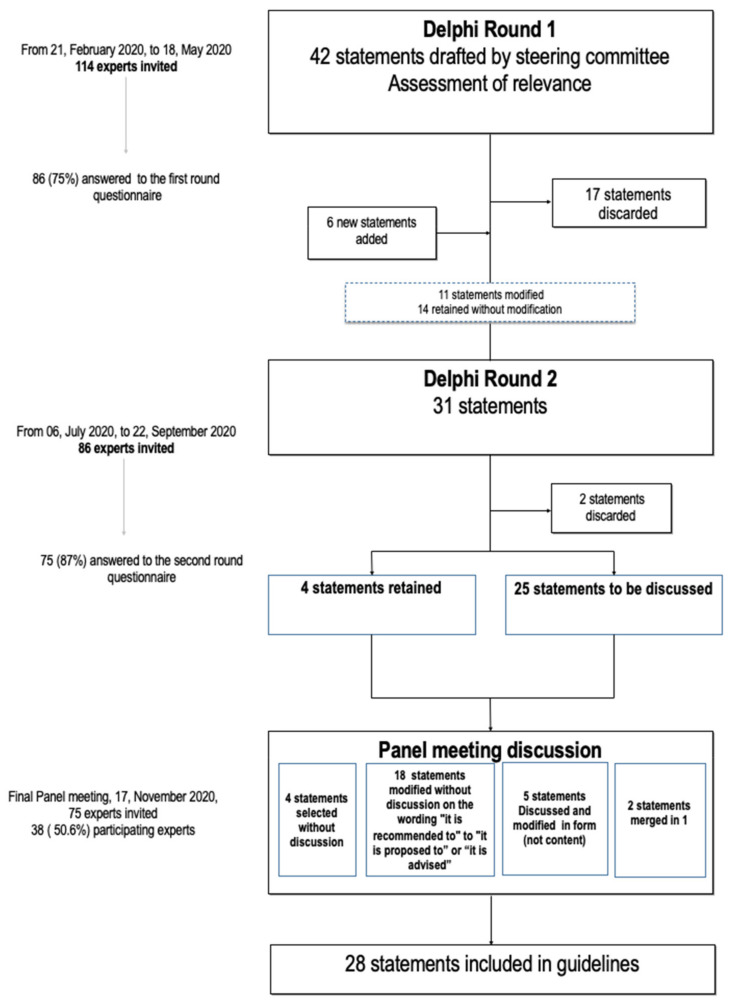
A stepwise two-round modified Delphi consensus survey to approve clinical guideline for fertility preservation in women with benign gynecologic disease.

Round 1 received responses from 75% of the stakeholders (86/114) (Figure 1). Data analysis resulted in the rejection of 17 statements and the selection of 14 without any modifications. Another 11 statements were modified based on comments from the respondents, who also proposed 6 new items that were included in the survey between Round 1 and 2. Two of these new questions were selected by the panel after Round 2.

#### 3.2.2. Delphi Round 2

The stakeholder response rate for Round 2 was 87% (75/86). Results led to the rejection of 2 of the remaining 31 statements.

#### 3.2.3. Approval of Selected Clinical Guidelines

Among the 86 participants asked to approve the final set of guidelines, 38 (50.6%) participated in the final videoconference to discuss and approve the final 29 statements. Comments led to the modification of the form, but not the substance, of five statements. Two statements were merged into one. Finally, a consensus approved 28 items, which form the final set of French clinical guidelines defining the indications for oocyte vitrification for fertility preservation in women with benign gynecologic disease (Table 2).

Table A1 presents the recommendations for which no consensus was reached. Experts rejected definitions of upper and lower thresholds to determine the cutoff age before or after which fertility preservation could not be offered. They also rejected a proposal to offer FP in rASRM stages I and II endometriosis without endometriomas.

## 4. Discussion

We present here the first guidelines focusing on FP in women with BGD after a scientifically designed Delphi process and with a high response rate by a large panel of health professionals and patients. Guidelines are at best derived from randomized controlled trials, which are ranked highest in the hierarchy of evidence (grade A) while expert opinion is ranked lowest in the evidence level (Grade D). However, the Delphi panel methodology allows survey of experts in a high quality and scientific manner. Level V evidence (expert opinion) remains a useful tool to determine the answer to a clinical question. The conclusions of the present study came from nationally recognized experts.

Stakeholders rated age-specific information concerning the risk of diminished ovarian reserve after surgery as important but rejected several upper and lower age limits. They determined that women must be informed about the benefit–risk balance of oocyte vitrification, in particular about the likelihood of live birth according to age at oocyte vitrification.

The ESHRE Guideline on Female Fertility Preservation does not distinguish BGDs from malignant conditions, given that personalized counseling about fertility preservation must be a systematic reflex by healthcare professionals before every gonadotoxic treatment, independent of its indication [2].

We have chosen to focus these guidelines on oocyte vitrification as an FP method. We voluntarily excluded statements about fertility-sparing surgical techniques during the Delphi questionnaire preparation, even though fertility-sparing gynecologic surgery would be of interest for specific guidelines [6,8].

### 4.1. Counseling Women of Reproductive Age with a Benign Gynecologic Disease

Counseling women before FP for benign indications was one of the major issues raised by the experts. Some stated that every woman should be warned before every operation associated with a risk of inducing a diminished ovarian reserve, such as ovarian cystectomy. Moreover, every woman should receive age-specific specialized information about the risks any ovarian stimulation and oocyte retrieval, with personalized counseling about the chances of live birth.

All studies agree about the need for age-specific information [9]: cryopreservation of an oocyte is not synonymous of FP, and the routine use of the ambiguous expression “fertility preservation” rather than “egg-freezing” may confuse women, often giving them false hopes of live births [10]. Our Delphi method results are thus consistent with the ESHRE guidelines, which also underline the importance of age-specific counseling in the light of women’s individual needs.

The risk of diminished ovarian reserve after ovarian surgery and the importance of the age at the time of oocyte cryopreservation are the main points that every gynecologic surgeon must know. The study by Cobo et al. (2018) of both oocyte survival rates after thawing and implantation rates showed a significantly higher cumulative live birth rate (CBLR) in women who had their oocytes cryopreserved before their 35th birthday [11]. For example, in non-malignant conditions, the CLBR with 15 vitrified oocytes is 69.8% for these women and only 38.8% afterwards.

The chances of live birth according to age at oocyte vitrification must be discussed together with the risks of the FP intervention. The principal risks of ovarian stimulation are OHSS and thrombosis. Grandone et al. reported a venous thromboembolism rate of 0.6% in women undergoing ART [12]. The odds ratio for such a venous thromboembolism among women undergoing ART who had been using estroprogestative contraception is higher, however—almost tripled (OR 2.96, 95% CI, 1.95–4.5). Accordingly, the overall risk in ART may not be the same as that for ovarian stimulation for BGD, especially among women using contraception, as women with endometriosis commonly do. The risks of oocyte retrieval are principally pelvic hemorrhage and pelvic postoperative infections, especially in women with endometriosis. The retrospective analysis of a cohort of 23,827 oocyte retrieval procedures conducted by Levi-Setti et al. (2018) estimated an overall complication rate of 0.4%. The overall risks of oocyte cryopreservation are low but must be balanced against the likelihood of CBLR. For example, Doyle et al. (2016) reported a livebirth rate of 2.5% per vitrified oocyte retrieved from women aged 41–42 years; this birth rate cannot justify the risk of the FP procedure [13].

### 4.2. Technical Aspect of Fertility Preservation for BGD

Experts endorsed only oocyte vitrification as an FP technique for BGD. This result is consistent with the ESHRE recommendations.

### 4.3. Indications for FP in Endometriosis

FP is a major concern for women with endometriosis, given the impact on their fertility of the disease and of the surgery required to treat it. The risk of diminished ovarian reserve after endometrioma surgery is well documented, and the indications for ovarian cystectomy have decreased [14]. Laparoscopic surgery might increase the pregnancy rate, but for now, no RCT has studied the live birth rate and the effect of laparoscopy on fertility remains uncertain [15]. If technically possible, the stakeholders advised ovarian stimulation first, before surgery for endometriosis. If the endometrioma is too large for easy retrieval, the experts advised surgical drainage rather than a cystectomy before ovarian stimulation. This recommendation is in line with the results of Cobo et al. (2018), who reported better ovarian response to ovarian stimulation and a significant better CLBR in women no older than 35 years without or before surgery (72.5%) compared with after surgery (52.8%). Consensus was difficult to reach for statement 19 as there is no single best procedure in the literature regarding the surgical drainage technique. Some experts perform simple drainage under GnRH agonists before ovarian stimulation for oocyte vitrification; others will prefer drainage with sclerotherapy before fertility preservation. As reported in the Appendix A showing the set of initial statements used in the Delphi process, the statement “Il is advised to perform sclerotherapy of endometriomas before ovarian stimulation for oocyte preservation” was discarded. Thus, in the statement 19, the term “drainage” alone may seem imprecise, but this is to leave practitioners free to carry out their usual protocol, pending more solid data from the literature.

Some authors would like to extend the FP indications for endometriosis when there is a high probability of IVF in the future, to freeze “younger oocytes” [5]. The participants in our study rejected this strategy on the grounds that FP should not be systematic for all women with endometriosis.

Cobo et al. (2020) also reported the observation of an egg-freezing program for 1044 women with endometriosis: among them 46.5% (*n* = 485) returned to use their vitrified oocytes and had a live birth rate of 46.4%, with 225 babies [16]. These women, however, returned for their vitrified oocytes only 1.5 years after vitrification, and 26.6% of the women who had not been pregnant with their returned frozen-thawed oocytes did finally become pregnant after IVF and fresh embryo transfer. Accordingly, we cannot reach a definitive conclusion about the real benefit of FP in endometriosis for obtaining a live birth compared to a first IVF strategy with a fresh embryo transfer [6].

The experts in our study also did not advise FP for stages I-II endometriosis without endometrioma. Rather, they recommended offering FP in women with endometrioma, and more specifically for bilateral endometriomas > 3 cm, recurrence after a first surgery for a unilateral endometrioma > 3 cm, and for endometrioma in a single ovary. In case of a first and single endometrioma > 3 cm, FP should be assessed case-by-case, taking age and ovarian reserve into account. The statement 17 stipulates that “When ovarian stimulation for fertility preservation is indicated for endometrioma(s), it is proposed to act if possible before cystectomy to increase the number of oocytes cryopreserved, if the ovaries are easily accessible for retrieval.” The aim of these guidelines was to provide guidance for practitioners to consider fertility preservation in the management of women with endometriomas. However, these guidelines were not intended to provide recommendations for the management of endometriosis. So, surgery or conservative treatment after FP will be decided on a case-by-case approach by the team that will take charge of it.

In a systematic review, however, Lantsberg et al. (2020) pointed out the lack of evidence concerning the effectiveness and long-term follow-up of FP for endometriosis. The interest of oocyte banking must be debated in endometriosis in the light of its potential medical risks and economic cost, given the high incidence of endometriosis in the general population, estimated at 6% to 10% of women of reproductive age [17].

### 4.4. Idiopathic Diminished Ovarian Reserve in the Absence of Gynecologic and Endocrinologic Diseases

The ESHRE guideline on Female Fertility Preservation does not recommend FP for women with overt primary ovarian insufficiency. In some pathologies, such as endometriosis or systemic lupus erythematosus, the relevance of pretreatment AMH levels for predicting the need for fertility preservation is unclear. The value of FP for women with reduced ovarian reserve is unclear, and ESHRE guideline recommend an individualized approach.

There are currently no data about the strategy for a fortuitous diagnosis of diminished ovarian reserve in healthy young woman. Oocyte cryopreservation is proposed for post-pubertal female children, adolescents, and young adults at risk of premature ovarian failure [3]. However, even in medical indications for FP, its efficacy, especially over the long term, is unknown [18].

Sometimes, young women of reproductive age are offered AMH testing for a “personalized fertility assessment” to discuss fertility preservation with the aim of postponing childbearing [19,20]. Anti-Mullerian hormone (AMH) is not a qualitative marker of fertility in healthy young women; spontaneous pregnancies are reported even for women with very low AMH levels [21]. AMH could, however, predict the age of menopause, especially for younger women, and a low AMH reflects a reduction in a woman’s reproductive lifespan, which might justify proposing FP [22,23]. Systematic FP in this indication is highly questionable, given the very limited data and the absence of long follow-up studies that could prove that this strategy would avoid unintended childlessness. Social egg-freezing could induce long-term disappointment, because women often overestimate their chance of pregnancy after oocyte cryopreservation [24].

The preferable strategy in response to a fortuitous diagnosis of diminished ovarian reserve remains to be determined. The experts in our study chose to not propose FP systematically either for women with a first-degree family history of premature ovarian insufficiency when their own ovarian reserve testing is normal or for a fortuitous diagnosis of idiopathic impairment of ovarian reserve. They specifically rejected ovarian tissue cryopreservation for this indication. Some authors propose this ovarian tissue cryopreservation with the aim of in vitro activation of ovarian cortex before autologous transplantation [25]. However, this innovative method must be interpreted cautiously and deserves further well-conducted studies.

## 5. Conclusions

To the best of our knowledge, we present here the first guidelines focusing on fertility preservation for women with benign gynecologic diseases and based on a scientifically designed Delphi process. These guidelines could be useful for gynecologists (i) to identify situations at risk of infertility, (ii) to provide better information for women with benign gynecologic diseases who might need personalized counseling for fertility preservation, and (iii) to standardize FP strategies for BGDs despite the current lack of an evidence base. However, cost-effectiveness and cost–benefit analyses are needed before concluding that egg banking is useful in the context of benign gynecologic diseases.

## Figures and Tables

**Table 1:** Characteristics of the PreFerBe expert panel members who participated in the Delphi survey.

	Round 1 (*n* = 86) *n* (%)	Round 2 (*n* = 75) *n* (%)
**Status**		
Physicians	80 (93)	72 (96)
Patients	6 (7)	3 (4)
Age (median) (Q1–Q3)	46 (37–54) (*n* = 81, 5 missing data)	46 (41–54) (*n* = 74, 2 missing data)
If physicians, years of experience (range)	17 (12–26) (*n* = 78, 2 missing data)	16.5 (12–25.25) (*n* = 74, 2 missing data)
**If physicians, specialty**		
Gynecology-obstetrics	54 (63)	46 (61)
Embryologist	16 (19)	16 (21)
Endocrinology	5 (6)	5 (7)
Radiology	3 (3)	3 (4)
Midwife	2 (2)	2 (3)
**If physicians, field of activity**		
Physician specialized in reproductive medicine	36 (45)	30 (40)
Gynecologic surgeons	20 (25)	19 (25)
Embryologist	15 (19)	15 (20)
Endocrinology	1 (1)	1 (1)
Other	5 (6)	4 (5)
Missing data	3 (4)	3 (4)
**If physicians, sector of activity**		
Public sector	48 (60)	44 (61)
Private sector	14 (18)	10 (14)
Public and private sectors	12 (15)	12 (17)
Missing data	6 (8)	6 (8)
If physicians, activity in a University Teaching Hospital	56 (70)	52 (72)
Participation in a learning society of the field	34 (39)	34 (45)

**Table 2:** Final set of French clinical guidelines defining indications for oocyte vitrification for fertility preservation in women with benign gynecologic disease.

**Counseling Women of Reproductive Age with Benign Gynecologic Disease about Fertility Preservation**	
1	Before any surgery at risk of ovarian damage, women of childbearing age should be informed of its potential effect on their ovarian reserve.
2	Women should be informed about the techniques for preserving their fertility most appropriate for them, according to their age and ovarian reserve.
3	Women should be informed that the use of the cryopreserved oocytes may never be necessary.
4	Women should be informed of the possible complications associated with ovarian stimulation and with oocyte retrieval.
5	Women should be informed that the use of fertility preservation techniques does not constitute a guarantee that they can have a child in the future.
6	Women should be informed of the objective chances of having a child after oocyte vitrification according to the number of vitrified oocytes and their age at the time of vitrification.
7	Women should be informed of the possibility of performing several cycles of stimulation to accumulate a sufficient number of oocytes.
8	Women should be given a reflection period to consider if they wish to commit themselves to the journey of fertility preservation.
9	A physician trained in reproductive medicine should inform the woman during a specific consultation about the techniques, modalities, results, and risks of fertility preservation, as well as of the regulatory conditions in effect in force.
10	Women with a benign gynecologic disease for which there is a risk that treatment might impair fertility should be informed about the desirable timeframe for implementing the appropriate fertility preservation procedures.
**Practical aspects of fertility preservation for benign gynecologic disease**	
11	Vitrification of mature oocytes after ovarian stimulation is the preferred method of fertility preservation for benign gynecologic disease.
**Indications for fertility preservation for endometriosis**	
12	Fertility preservation should be proposed for bilateral endometriomas > 3 cm.
13	It is not advised to propose fertility preservation for a first episode of unilateral endometrioma < 3 cm in a woman with an ovarian reserve normal for her age.
14	For a first episode of unilateral endometrioma > 3 cm, it is advised to assess the indication for fertility preservation on a case-by-case basis according to age and ovarian reserve.
15	It is proposed to discuss fertility preservation for a recurrent unilateral endometrioma.
16	It is advised to propose fertility preservation for an endometrioma on a single ovary.
17	When ovarian stimulation for fertility preservation is indicated for endometrioma(s), it is proposed to act if possible before cystectomy to increase the number of oocytes cryopreserved, if the ovaries are easily accessible for retrieval.
18	It is not advised to propose fertility preservation for minimal to mild endometriosis that does not affect the ovaries.
19	When ovarian stimulation for fertility preservation is indicated for endometrioma(s), drainage should be performed in first line if the endometriomas are too bulky and/or if they prevent easy access to the ovaries for retrieval.
**Other indications for fertility preservation for benign gynecologic disease: tubal disease, persistent ovarian cysts, and fibroids**	
20	It is not advised to propose fertility preservation before surgery for a first persistent unilateral non-endometriotic ovarian cyst episode.
21	It is proposed to discuss fertility preservation if surgery is indicated for bilateral persistent ovarian cysts, depending on age and ovarian reserve.
22	Fertility preservation is not proposed for isolated uterine adenomyosis.
23	It is proposed to discuss fertility preservation if surgery is indicated for presumed benign persistent ovarian cyst(s) on a single ovary.
24	It is proposed to discuss fertility preservation if surgery is indicated for recurrent benign persistent ovarian cyst(s), depending on age and ovarian reserve.
25	It is not advised to propose fertility preservation for isolated fibromatous disease.
26	In the case of surgery for benign gynecologic disease at presumed risk of impaired ovarian function, preoperative ovarian reserve testing is proposed.
**Fertility preservation for idiopathic ovarian reserve in the absence of gynecologic and endocrinologic diseases**	
27	For women with a first-degree family history of premature ovarian insufficiency, it is advised to perform regular follow-up of their ovarian reserve to be able to propose fertility preservation if necessary.
28	Should a substantial impairment of ovarian reserve for age be discovered fortuitously and indicate the need for an etiological workup, it is proposed to discuss fertility preservation on a case-by-case basis.

## Data Availability

All the data are presented in this study.

## Appendix A

**Table A1:** Set of initial and final statements used in the Delphi process to define indications for fertility preservation in women with benign gynecologic disease, with a description of the selection process through the approval of the final clinical guideline.

Round 1				Round 2				Panel Meeting Discussion
Initial Proposed Items	Median	% ≥ 7	Status	Modified Formulation (if Applicable)	Median	% ≥ 7	Status	Consensus Formulation of the Final Retained Items
**Counseling women of reproductive age with benign gynecologic disease about fertility preservation**								
Before any surgery with a risk of ovarian damage, women of childbearing age must be informed of its potential effect on their ovarian reserve.	9	95%	Modified	Before any surgery with a presumed risk of ovarian damage, women of childbearing age must be informed of its potential effect on their ovarian reserve.	9	99%	Modified	Before any surgery at risk of ovarian damage, women of childbearing age should be informed of its potential effect on their ovarian reserve.
Women must be informed about the different techniques for fertility preservation.	9	83%	Modified	Women must be informed about the techniques for fertility preservation most appropriate for them, according to their age and ovarian reserve.	9	93%	Modified	Women should be informed about the techniques for preserving their fertility most appropriate for them, according to their age and ovarian reserve.
Women must be informed that the reuse of the preserved gametes may never be necessary.	8	91%	Retained		9	95%	Modified	Women should be informed that the use of the cryopreserved oocytes may never be necessary.
Women must be informed of the possible complications associated with ovarian stimulation and with oocyte retrieval.	9	86%	Retained		9	96%	Modified	Women should be informed of the possible complications associated with ovarian stimulation and with oocyte retrieval.
Women must be informed that fertility preservation techniques do not constitute a guarantee that they can have a child in the future.	9	97%	Retained		9	99%	Modified	Women should be informed that the use of fertility preservation techniques does not constitute a guarantee that they can have a child in the future.
Women must be informed of the objective chances of having a child after oocyte vitrification according to the number of vitrified oocytes and their age at the time of vitrification.	9	86%	Retained		9	96%	Modified	Women should be informed of the objective chances of having a child after oocyte vitrification according to the number of vitrified oocytes and their age at the time of vitrification.
It is advised that women be informed of the possibility of performing several cycles of stimulation to accumulate a sufficient number of oocytes.	9	87%	Retained		9	95%	Modified	Women should be informed of the possibility of performing several cycles of stimulation to accumulate a sufficient number of oocytes.
It is advised to give women a waiting period to decide if they wish to launch themselves into the journey of fertility preservation.	9	90%	Modified	It is advised to give women a waiting period to decide if they wish to commit themselves to the journey of fertility preservation.	9	95%	Modified	Women should be given a reflection period to consider if they wish to commit themselves to the journey of fertility preservation.
Women who are candidates for fertility preservation must be informed of the legal and administrative conditions in force.	9	88%	Retained		9	93%	Modified and merged	A physician trained in reproductive medicine should inform the woman during a specific consultation about the techniques, modalities, results, and risks of fertility preservation, as well as of the regulatory conditions in effect in force.
/	/	/	Added	A consultation with a specialist in reproductive medicine must take place to explain the techniques, modalities, results, and risks of fertility preservation.	9	96%		
/	/	/	Added	In the case of benign gynecologic disease for which there is a risk that treatment might impair fertility, women must be informed about the conditions of access to fertility preservation and time required for it.	9	95%	Modified	Women with a benign gynecologic disease for which there is a risk that treatment might impair fertility should be informed about the desirable timeframe for implementing the appropriate fertility preservation procedures.
**Practical aspects of fertility preservation for benign gynecologic disease**								
It is advised to prefer vitrification of mature oocytes after ovarian stimulation.	9	97%	Retained		9	97%	Modified	Vitrification of mature oocytes after ovarian stimulation is the preferred method of fertility preservation for benign gynecologic disease.
It is advised to propose 37 years as the maximum age at which oocyte preservation should be offered.	5	25%	Discarded	/	/	/	/	/
It is advised to propose 40 years as the maximum age at which oocyte preservation should be offered.	6	40%	Discarded	/	/	/	/	/
It is not advised to perform fertility preservation for benign gynecologic disease when the biomarkers (FSH and blood estradiol at the beginning of the follicular phase, and AMH) and ultrasound (antral follicle count) already show a severely diminished ovarian reserve.	5	33%	Discarded	/	/	/	/	/
It is advised to await the age of 23 years before proposing oocyte cryopreservation because of the higher risk of oocyte aneuploidy among very young women.	4	25%	Discarded	/	/	/	/	/
It is advised to await if possible the age of 18 years before proposing oocyte cryopreservation because of the higher risk of oocyte aneuploidy among very young women.	5	35%	Discarded	/	/	/	/	/
**Indications for fertility preservation for endometriosis**								
It is advised to propose fertility preservation for bilateral endometriomas > 3 cm.	9	79%	Retained		9	85%	Modified	Fertility preservation should be proposed for bilateral endometriomas > 3 cm.
It is advised to propose fertility preservation for voluminous unilateral endometrioma.	7	52%	Discarded	/	/	/	/	/
It is advised to propose fertility preservation for unilateral endometrioma ≥ 6 cm.	7	53%	Discarded	/	/	/	/	/
It is not advised to envision fertility preservation for a first episode of unilateral endometrioma < 3 cm.	8	65%	Modified	It is not advised to envision fertility preservation for a first episode of unilateral endometriomas < 3 cm in a woman with an ovarian reserve normal for her age.	8	84%	Modified	It is not advised to propose fertility preservation for a first episode of unilateral endometrioma < 3 cm in a woman with an ovarian reserve normal for her age.
In the case of a first episode of unilateral endometrioma between 3 and 6 cm, it is advised to assess the indication for fertility preservation on a case-by-case basis according to age and ovarian reserve.	9	77%	Retained		9	89%	Modified	For a first episode of unilateral endometrioma > 3 cm, it is advised to assess the indication for fertility preservation on a case-by-case basis according to age and ovarian reserve.
It is advised to propose fertility preservation for multiple endometriomas > 3 cm on the same ovary.	7	63%	Discarded	/	/	/	/	/
It is advised to propose fertility preservation for a recurrent unilateral endometrioma.	8	79%	Retained		8	88%	Modified	It is proposed to discuss fertility preservation for a recurrent unilateral endometrioma.
It is advised to propose fertility preservation for an endometrioma on a single ovary.	9	82%	Retained		9	88%	Modified	It is advised to propose fertility preservation for an endometrioma on a single ovary.
For a woman with no immediate plans to have a child, it is advised to propose fertility preservation if she had endometriosis that will require IVF should she want a child in the future.	8	61%	Discarded	/	/	/	/	/
When it is decided that fertility preservation is indicated for endometrioma(s), it is advised to act if possible before surgery to increase the number of oocytes preserved.	8	71%	Modified	When ovarian stimulation for fertility preservation is indicated for endometrioma(s), it is advised to act if possible before surgery to increase the number of oocytes preserved, if the ovaries are easily accessible for retrieval.	8	85%	Modified	When ovarian stimulation for fertility preservation is indicated for endometrioma(s), it is proposed to act if possible before cystectomy to increase the number of oocytes cryopreserved, if the ovaries are easily accessible for retrieval.
It is advised to perform sclerotherapy of endometriomas before ovarian stimulation for oocyte preservation.	5	24%	Discarded	/	/	/	/	/
It is not advised to propose fertility preservation for minimal to mild endometriosis.	8	71%	Modified	It is not advised to propose fertility preservation for minimal to mild endometriosis that does not affect the ovaries.	8	89%	Retained	It is not advised to propose fertility preservation for minimal to mild endometriosis that does not affect the ovaries.
It is not advised to propose fertility preservation for deep endometriosis with no tubal or ovarian damage.	7	51%	Discarded	/	/	/	/	/
/	/	/	Added	When ovarian stimulation for fertility preservation is indicated for endometrioma(s), it is advised to perform it after drainage if the endometriomas are too bulky and/or prevent easy access to the ovaries for retrieval.	8	82%	Modified	When ovarian stimulation for fertility preservation is indicated for endometrioma(s), drainage should be performed in first-line if the endometriomas are too bulky and/or if they prevent easy access to the ovaries for retrieval.
**Other indications for fertility preservation for benign gynecologic disease: tubal disease, persistent ovarian cysts, fibroids**								
It is advised to propose fertility preservation in the case of severe tubal impairment for which IVF will be probably necessary if pregnancy should be desired.	5	36%	Discarded	/	/	/	/	/
It is not advised to propose fertility preservation before surgery for a first persistent unilateral non-endometriotic ovarian cyst episode.	8	76%	Retained		8	88%	Retained	It is not advised to propose fertility preservation before surgery for a first persistent unilateral non-endometriotic ovarian cyst episode.
It is advised to discuss fertility preservation for a first bilateral persistent ovarian cyst episode.	8	73%	Modified	It is advised to discuss fertility preservation if surgery is indicated for bilateral persistent ovarian cysts, depending on age and ovarian reserve.	8.5	89%	Modified	It is proposed to discuss fertility preservation if surgery is indicated for bilateral persistent ovarian cysts, depending on age and ovarian reserve.
Fertility preservation must not be proposed for isolated uterine adenomyosis.	8	86%	Retained		9	94%	Modified	Fertility preservation is not proposed for isolated uterine adenomyosis.
After adnexal torsion, it is advised to discuss fertility preservation on a case-by-case basis.	7	58%	Discarded	/	/	/	/	/
Fertility preservation must not be proposed in the case of a single ovary with no disease at risk of diminished ovarian reserve associated with the procedure.	7	56%	Discarded	/	/	/	/	/
Fertility preservation must be proposed in cases of presumed benign persistent ovarian cyst(s) on a single ovary.	7	67%	Modified	It is advised to discuss fertility preservation if surgery is indicated for presumed benign persistent ovarian cyst(s) on single ovary, depending on age and ovarian reserve.	8	90%	Modified	It is proposed to discuss fertility preservation if surgery is indicated for presumed benign persistent ovarian cyst(s) on a single ovary.
Fertility preservation must be proposed after surgery for a recurrent persistent ovarian cysts presumed to be benign.	8	73%	Modified	It is advised to discuss fertility preservation if surgery is indicated for a recurrent benign persistent ovarian cyst(s), depending on age and ovarian reserve.	9	92%	Modified	It is proposed to discuss fertility preservation if surgery is indicated for recurrent benign persistent ovarian cyst(s), depending on age and ovarian reserve.
Fertility preservation must not be proposed for isolated fibromatous disease.	8	81%	Retained		8	89%	Modified	It is not advised to propose fertility preservation for isolated fibromatous disease.
Fertility preservation is advised when embolization of uterine fibromas is indicated in a woman of childbearing age.	5	36%	Discarded	/	/	/	/	/
/	/	/	Added	In the case of surgery for benign gynecologic disease at presumed risk of impaired ovarian function, preoperative ovarian reserve testing is proposed.	9	90%	Retained	In the case of surgery for benign gynecologic disease at presumed risk of impaired ovarian function, preoperative ovarian reserve testing is proposed.
/	/	/	Added	In the case of surgery for persistent benign ovarian cysts except for endometrioma (dermoid, seromucinous, etc.) when fertility preservation is indicated, it is advised to proceed to oocyte preservation after ovarian surgery.	7	55%	Discarded	/
/	/	/	Added	When embolization of uterine fibromas is indicated as an alternative to hysterectomy, it is proposed that oocyte preservation be discussed as a function of age and ovarian reserve.	7	51%	Discarded	/
**Fertility preservation for idiopathic ovarian reserve in the absence of gynecologic and endocrinologic diseases**								
It is advised to discuss fertility preservation for women with a first-degree family history of premature ovarian insufficiency.	7	67%	Modified	For women with a first-degree family history of premature ovarian insufficiency, it is advised to perform regular follow-up of their ovarian reserve to be able to propose fertility preservation if necessary.	8	82%	Retained	For women with a first-degree family history of premature ovarian insufficiency, it is advised to perform regular follow-up of their ovarian reserve to be able to propose fertility preservation if necessary.
It is advised not to propose cryopreservation of ovarian tissue for a woman referred for consultation about fertility preservation for a diminished ovarian reserve.	7	55%	Discarded	/	/	/	/	/
Should an abnormally diminished ovarian reserve be discovered fortuitously, it is advised to discuss fertility preservation on a case-by-case basis in cooperation with geneticists.	8	78%	Modified	Should a severe impairment of ovarian reserve for age be discovered fortuitously and indicate the need for an etiological workup, it is advised to discuss fertility preservation on a case-by-case basis as a function of the results of the genetic workup.	9	89%	Modified	Should a substantial impairment of ovarian reserve for age be discovered fortuitously and indicate the need for an etiological workup, it is proposed to discuss fertility preservation on a case-by-case basis.
Should a diminished AMH level be discovered fortuitously a single time, it is not recommended to propose fertility preservation.	6	42%	Discarded	/	/	/	/	/
